# Enhancing English as a foreign language university students’ writing through explicit instruction of conjunctions as cohesive devices: An experimental study

**DOI:** 10.3389/fpsyg.2022.1053310

**Published:** 2022-11-28

**Authors:** Arwa Saleh Alawerdy, Fahd Shehail Alalwi

**Affiliations:** Department of English, College of Science and Humanities, Prince Sattam Bin Abdulaziz University, Al-Kharj, Saudi Arabia

**Keywords:** EFL, ESL, cohesive devices, conjunctions, paragraph writing, TESOL

## Abstract

Cohesion has long been regarded as one of the main components of successful writing. The basic assumption of this concept is that the interpretation of one element in a text is based on the interpretation of another in the same text. It has been noticed that Saudi EFL students seem to lack some basics in writing a cohesive paragraph. Certain conjunctions were overused, underused, or misused. The present empirical study, therefore, attempted to investigate the impact of explicit teaching of conjunctions as cohesive devices on the development of paragraph writing. The subjects of this study were 43 EFL students at a Saudi University studying in the first year. Data were collected from pre and post tests and analyzed qualitatively and quantitatively. Results showed significant differences between the experimental and control groups in the post test. Pedagogical implications are presented.

## Introduction

In the context of teaching English as a foreign language (EFL) and English as a second language (ESL), one of the main language skills is writing. Developing writing skills is regarded to be very complicated to both EFL learners and teachers. It was found that in their writings, learners found difficulties in arranging their ideas logically (Khelifii, 2014). Holloway (1981) maintained that students should be urged to ensure that their content flows through a succession of phrases while conveying meaningful concepts in writing. As a result, it is critical to steer them to the concepts they intend to communicate as well as words that effectively express their thoughts.

The challenges that are encountered by EFL/ESL students when writing have been the subject of numerous studies recently. Cohesion, in particular, has received considerable attention. A significant amount of research on ESL and EFL writing has been based on Halliday and Hasan’s (1976) approach to cohesion and coherence. In this regard, Halliday and Hasan (1976) argued that cohesiveness and coherence, as two fundamental textual aspects, have long been recognized as important characteristics of effective writing. As a result, language learners must invariably compose clear and cohesive writings if they want to demonstrate that they are qualified English writers. It is therefore important for EFL teachers to promote cohesion and cohesive devices in their students’ writings, as this is key to writing logically arranged paragraphs.

In teaching writing in the Saudi Arabian context where English is the medium of instruction in higher education, the majority of teachers were found to be more concerned with the sentence level instead of the discourse level (Alshalan, 2019). This finding suggests that cohesiveness is only marginally dealt with in the Saudi EFL classroom. In her study, Alshalan (2019), p.49 recommended that teachers focus more on raising learners’ attention to the usage of cohesive devices and provide more hands-on training sessions to their students.

It has been observed that EFL students in their first year of study in Saudi Arabia lack some basics in writing a cohesive paragraph. They tend to overuse, underuse, and misuse certain conjunctions. The present study sought to contribute to finding a strategy that could enhance students’ knowledge and develop their writing skills. Accordingly, the main purpose of this investigation was to examine whether explicit instruction of conjunctions as cohesive devices affects EFL students’ paragraph writing abilities.

The present study is administered at the Preparatory Year Program (PYP), Prince Sattam bin Abdulaziz University, Saudi Arabia. It is the first year in which all students take the same courses. A key purpose of this program is to enable students to smoothly transition from high school at which Arabic is the medium of instruction to higher education where English is the medium of instruction, with emphasis on enhancing English language skills. There are two tracks in PYP: the health colleges track and the science and engineering colleges track. The former leads to a college of medicine, dentistry, pharmacy, or applied medical sciences. As for the latter, it leads to a college of engineering, computer science and engineering, or a college of sciences (Alalwi, 2021). Yee Siu and Afzaal (2022) underscored the importance of writing for entrants in the engineering market. In the present study, 43 female students enrolled in the engineering track participated in order for us to investigate the following research questions:

(1) What is the impact of the explicit instruction of the conjunctions as cohesive devices on Saudi university EFL students’ paragraph writing performance?
(2) Do the post-test results of the experimental group and control group show any statistically significant difference?

Prior to identifying the materials and methods that were used in this study, a closer look at the definition of paragraph writing and the cohesion theory is briefly introduced followed by a review of previous studies.

Creating an essay requires students to understand how words are used to construct sentences, sentences to construct paragraphs, and paragraphs to construct essays (Folse et al., 2020). Boukra et al. (2019) suggested that writers must adhere to certain stages in order to develop and articulate their ideas properly before presenting them to the reader. These include several operations such as brainstorming, outlining, preparation, organization, and revision. Generally, writing is considered a challenging skill. It is a complex skill, even in one’s native language, hence, EFL learners face additional difficulties. Many English teachers have observed that learning a skill such as writing is more challenging than learning any other language skill (Rassouli and Abbasvandi, 2013). Furthermore, Alsmari (2019) stated that it takes mental effort to plan the sentences and connect them to make writing meaningful and communicative.

A paragraph is a fundamental component of all written composition as maintained by Kolin (2001), who described the paragraph as a set of connected sentences organized logically to give readers comprehensive information about a specific topic. Basically, a paragraph consists of linked sentences that address a single topic. The length depends on the topic, obviously, but it must be sufficiently developed to support the main idea. In this regard, Oshima and Hogue (2006) suggested that the paragraph could be composed of one or ten sentences. It can be said then that the paragraph length is not significant; it should, however, illustrate the main idea. In addition, there is a specific way that a paragraph is organized. The logical structure of a paragraph allows readers to follow the writer’s thoughts Zemach and Rumisek (2003) indicated that a proper paragraph should begin with a clear topic sentence which is supported with background information and some details, and should end with a concluding sentence.

Boukra et al. (2019) stated that effective paragraphs are characterized by being unified, coherent, cohesive, complete, and varied. Writers are, therefore, advised to build their sentences that are related to the main topic. Each of these sentences must flow smoothly within the text, and the sentences should be connected through different cohesive devices. Writers should develop their ideas in a complete way to avoid any dissatisfaction about the quality and quantity of the writing. Moreover, writers are encouraged to use various types of sentences in order to show more varied and unique way of writing. Nevertheless, cohesion is of paramount importance (Du et al., 2022).

The concept of cohesion originated from Halliday and Hasan (1976) theory. Apparently, the premise of cohesion is not that the text is merely a unit of form, but also that it is a reflection of meaning relations within the text. As such, the text is regarded as a semantic unit that shows dependency between the interpretations of elements in a discourse. In order to be able to interpret these semantic relationships within and across sentences, readers must take into account all the other contextual details contained in that text. According to Halliday and Hasan (1976), cohesion requires employing cohesive devices that fall under two categories: lexical and grammatical. Lexical cohesive devices are based on the meaning of words, rather than their form, as opposed to grammatical cohesive devices, which emphasize the relationship between sentences. Table 1 summarizes Halliday and Hasan’s (1976) classification of cohesion and its subcategories. Since the present study’s main objective is to examine the role of conjunctions and the explicit instruction thereof on EFL learners’ writing development, a detailed explanation of conjunctions will be provided.

**TABLE 1:** Types of Cohesion based on Halliday and Hasan (1976) (Adapted from Tsareva, 2010, p. 10).

Cohesion				
	
Grammatical			Lexical	
Reference	Exophoric [situational]		Reiteration	Repetition
	Endophoric [textual]			Synonyms
	Anaphoric [to preceding text]	Cataphoric [to following text]		Superordinate
Ellipsis				General word
Substitution			Collocation	
Conjunction				

Halliday and Hasan (1976) observed that conjunctions express some cohesive relation in an indirect way through certain meanings. Other components of the discourse are presupposed by these meanings. Thus, they treated conjunctive elements as cohesive ties. Conjunction, as they suggested, is a type of cohesion in which sentences in a text are linked together using a set of ties. The conjunction defines a relationship between two ideas in a discourse; understanding the first idea is essential to understanding the second, and reflect the logical-semantic relationship across sentences in a text (Halliday and Hasan, 1976). Put simply, using conjunctions can help writers in organizing the content in a way that the readers can understand. In Halliday and Hasan’s (1976) framework for categorizing conjunctive relations, four categories are described: additive, adversative, causal, and temporal, each category is further subdivided. The categories and subcategories of each type of conjunction are shown in Table 2.

**TABLE 2:** Halliday and Hasan’s classification of conjunction (Adapted from Tsareva, 2010, p.31).

Types of conjunctions			
Additive	Adversative	Causal	Temporal
Simple: and, nor, or	Proper: yet, but, however	General: so, because of, thus	Simple: then, next, afterward
complex: moreover, in addition, besides that, additionally	Contrastive: but, on the other hand, in fact, actually, at the same time	Specific: for this reason, as a result, for this purpose	Complex: at once, this time, the last time, meanwhile, at this moment, until then
Comparative: likewise, similarly, on the other hand	Corrective: instead, on the contrary, at least	Conditional: then, under the circumstances	Sequential/conclusive: at first, in the end; finally, at last
Appositive: I mean, in other words, for example, thus	Dismissive: in any case, anyhow, at any rate	Respective: in this respect, with regard to this, otherwise	“Here and now”/summarizing: up to now, up to this point; to sum up, briefly

One of the most extensively explored subfields of second language writing is cohesion. However, Saudi EFL learners are still underrepresented (Alshalan, 2019). Needless to say, Halliday and Hasan’s (1976) framework has been widely adopted in research on cohesion and coherence in ESL/EFL. Although several researchers reached similar conclusions, in some cases the results of these studies were quite contradictory. Several studies have found no difference between good and bad manuscripts in terms of the use of cohesive devices (Al Shamalatm and Ghani, 2020). On the contrary, Alshalan (2019) found that the use of cohesive devices is one sign of high-quality texts. Her study indicated that high-scoring essays seemed to have more cohesive devices than low-scoring ones.

Alqasham et al. (2021) conducted a study to look at the coherence and cohesiveness of Saudi EFL tertiary students’ essay writing. To investigate the cohesive devices, they included both quantitative and qualitative tools. As part of the study, the written work of 50 Saudi EFL students at Qassim University served as the corpus of the study. They applied Halliday and Hasan’s (1976) framework of cohesion to analyze the essays. Frequency counts were used to monitor the students’ use of cohesive devices. They found out that students’ performance was typically low. It was also noticed that students lacked the ability to employ cohesion in their writings. They suggested that rather than depending just on accurate spelling and grammar, EFL students can undertake continuous writing exercises to enhance their writing abilities and create effective texts (Alqasham et al., 2021).

In the same vein, Alshalan (2019) conducted a study in an attempt to examine how often the cohesive devices were used by students, along with their association with the writing quality. One hundred EFL students at Al Imam Muhammed Ibn Saud Islamic University were included in her study. Alshalan employed a mixed methods approach in which the students’ essays were studied in terms of the textual meta-function of cohesive devices by using systemic functional linguistics (SFL). The results of her study indicated that Saudi students tend to frequently use repetition in their writing. Moreover, she found out that there were positive correlations between the writing scores of the students and the length of their essays. In addition, there were positive correlations between the scores and the use of cohesive devices, as well as between the length and the use of cohesive ties.

Most recently, a study was conducted in Sudan by Yousif (2021) who sought to examine the extent to which EFL learners might employ cohesive devices. A test on cohesive devices was given to 150 students. The study employed an analytical descriptive method to evaluate the test, and the data was analyzed using SPSS. in her study, Yousif (2021) found out that nearly 60% of students passed the exam, while 40% failed. She concluded that cohesive devices continue to be a source of difficulty for many EFL students.

While the studies above explored both types of cohesive ties in students’ writing, other researchers focused on one type or even a subtype. A study carried out by Abdelreheim (2014) examined the use of grammatical cohesive devices by Emirati eighth-grade EFL learners in expository texts. He employed Halliday and Hasan’s (1976) grammatical cohesion model to evaluate a sample of 30 written expositions produced by learners. A mixed-methods study was performed to determine the most commonly used device based on percentages and numbers, and to identify problems that learners had when working with these devices. In his study, Abdelreheim (2014) found out that all types of grammatical cohesion were employed: references, substitutions, ellipses, and conjunctions. Conjunctional devices were largely used followed by references, while elliptic and substitutional were rarely employed.

Aziz and Nuri (2021) studied the use of conjunctive adverbs in essays written by Iraqi Kurds EFL learners. In order to find out which types of conjunctive adverbs are overused, underused, or misused and in which position in sentences, they created a corpus of 50 students’ writings and analyzed the data quantitatively using AntConc, a corpus analysis software. The results showed that students used more sequential and additive conjunctive adverbs than adversatives and causative adverbs. Aziz and Nuri (2021) also found that learners relied mainly on conjunctive adverbs in the initial position.

In their study of English major Jordanians, Al Shamalatm and Ghani (2020) conducted a study to examine the effect of conjunction use on overall writing quality. They employed a quantitative design to examine how cohesive devices, specifically conjunctions, are used in essays that are written argumentatively by sixty Jordanian English majors. They used written essays and interviews to collect data, which was then analyzed with SPSS. The findings of their study revealed that using conjunctions as cohesive devices had a weak negative but insignificant correlation with writing quality. This suggests that students’ regular use of such devices in their writing had no effect on the quality of their writing under any circumstances.

Additionally, some peculiar features have been observed in the writing of ESL/EFL students. As part of a study conducted in Tabuk, Saudi Arabia, Othman (2019) examined the grammatical cohesive devices errors committed by students and the types, frequencies and causes of such errors. He found that the students repeatedly committed errors related to the use of a conjunction as a cohesive device. Othman (2019) further added that such errors were committed due to several factors including the students’ lack of knowledge of such devices, their poor vocabulary, the mother tongue interference, and the language background. In the same regard, Arabi and Amin Ali (2014) investigated Sudanese students’ use of the cohesion subset of conjunctions. The results revealed that the majority of errors were created by additives, followed by causals and adversatives. They assumed that students transferred some characteristics from their mother tongue into English. However, Adiantika (2015) found out in his study EFL students that their errors were due to the lack of training on the appropriate use of cohesive devices.

The previous studies reviewed above have shown that conjunctions are important for writing and that EFL learners have considerable difficulty applying them. Research suggests that EFL instructors can use several approaches to motivate students to learn cohesion and to be better writers. Providing more detailed explanations of how to use cohesion is one example. This will help minimize mistakes made by students when they write a text in the future (Abdelreheim, 2014; Adiantika, 2015; Othman, 2019; Alqasham et al., 2021). A study conducted by Boukra et al. (2019) examined the effectiveness of explicit instruction of grammatical cohesive devices on students’ paragraph writing quality by adopting a quasi-experimental design to compare compositions produced by students who had instruction to compositions produced by students who did not. The study samples consisted of forty students at L’Arbi Ben Mhidi University who were enrolled in the EFL program for the first year. They were equally divided into two groups. Based on the quantitative analysis of the data, it was verified that the instruction improved the writing quality of the students.

The present study employed the explicit instruction of conjunctions as a means to enhance the EFL students’ paragraph writing, and the context is explained below.

## Materials and methods

### Design

In order to investigate the effectiveness of explicit instruction of conjunctions as cohesive ties in developing paragraph writing skills in Saudi EFL students, an experimental and control group design with a pre- and post-test was adopted. Before the experiment began, both groups were required to have a paragraph writing test as well as a written diagnostic test. Then, the experimental group underwent an intervention where instruction of conjunctions was employed whereas the control group studied the same course material without any additional intervention.

### Participants

The participants of this study were 43 EFL students at Prince Sattam bin Abdulaziz University studying at the preparatory year program (PYP), Saudi Arabia. One of the main courses is Writing Skills Eng1220, which aimed at enhancing students’ writing abilities in the target language. In the present study, participants were selected randomly from two classes and divided into two groups: an experimental group (*n* = 21) and a control group (*n* = 22). Further, to ensure homogeneity and comparability in terms of English language proficiency between the two groups, participants underwent an Oxford Placement Test (OPT).

### Instruments

#### Oxford placement test

All participants took the Oxford Placement Test (OPT) at the beginning of the study. Students at PYP are required to take this test to determine their English proficiency level. Based on the Common European Framework of Reference (CEFR) (Council of Europe, 2020), results demonstrated that nearly 71% of the participants were English basic users or lower with 8% of the students are starters (Pre-A), 37% are beginners (A1), 26% elementary learners (A2), 15% lower intermediate learners (B1), 8% upper intermediate learners (B2), and 4% advanced (C).

#### Written diagnostic test

A written diagnostic test on conjunctions, adopted from Yousif (2021), was administered pre and post the instruction. This test measures the students’ competence regarding conjunction. It has three sections. The first section is a completion question concerning different types of conjunctions, the second question consists of ten connectors and their functions to be identified, whereas the third one is a multiple-choice question.

#### Paragraph writing test

The students in both groups were asked to write a paragraph of about 100–150 words before and after the intervention in order to compare and assess the effectiveness of the instruction on the students’ writings. This test measures the students’ ability to use conjunctions appropriately in their writing, and how such cohesive devices contribute to developing their performance. A writing rubric was adopted from Abdelreheim (2014) for this purpose.

### Procedures

To elaborate more on what has been mentioned above, the present study was conducted in three stages: pre-tests, intervention, and post-tests. First, in order to ensure the groups’ homogeneity, both groups were required to undergo pre-tests to measure their ability to compose cohesive paragraphs and to assess their knowledge regarding conjunctions as cohesive devices as mentioned above. Second, the intervention was applied exclusively to the experimental group. As for the control group, there was no explicit instruction. Finally, post-tests were administered to determine whether such intervention had any significant difference.

As for the intervention, the experimental group students received five oral sessions on conjunctions, one session per week. Each session lasted 30 min. In the first session there was in introduction to the concept of cohesion and its different grammatical and lexical types. For the remaining four sessions, students were given a detailed explanation of conjunctions in particular, including their types, meanings, functions, and uses. The objective of the treatment was that by the end of these sessions, students would be able to understand all types and subtypes of conjunctive devices, and they would be able to use them appropriately.

Moreover, lessons were enriched with hands-on training to enhance students’ relevant skills. For instance, students were required to read a passage and identify additive conjunctive ties. Also, they were required to connect two sentences using various devices. In another activity, they were asked to use temporal conjunctions in a story they would narrate.

Halliday and Hasan’s (1976) taxonomy of conjunctions was used as a basis for developing the lessons as it provides a clear and comprehensive overview of conjunctions. (Shown in Table 2).

### Data analysis

Data were analyzed qualitatively and quantitatively. The results of pre- and post-WDT were analyzed quantitatively using *t*-test with SPSS. As for the PWT, paragraphs were analyzed qualitatively as frequencies of conjunctions were counted and their appropriateness was evaluated by a panel of experts using the rubric mentioned above.

## Results and discussion

Aiming to improve EFL students’ writing, this study explored the potential benefits of teaching conjunctions explicitly. Using Halliday and Hasan’s (1976) conjunctive framework, this study analyzed students’ use of cohesive devices before and after intervention. A combination of quantitative and qualitative methods was used to analyze the pre- and post-tests data. Moreover, in order to have a well-rounded understanding of students’ performance, samples of their writings are provided below where necessary.

### Results of written diagnostic test

#### Pre-test

Before any intervention, a pre- WDT was administered to all participants to ensure that both groups were on the same level and had equivalent knowledge of conjunctions. An independent samples *t*-test was used to assess whether the control group (*n* = 22) and the experimental group (*n* = 21). Based on the result shown in Table 3, both groups had almost identical mean scores in the pre-test (11.1 and 9.5, respectively) regarding their knowledge of connectors. In this case, the *p*-value detected (*p* = 0.314) is greater than the pre-determined significant level (*p* < 0.05). Hence, the values obtained for both groups revealed that there was no statistically significant difference prior to the intervention.

**TABLE 3:** Independent samples *t*-test for the difference in the mean scores of (WDT) pre-test for the experimental group and the control group.

Group	Number	Mean	Std. deviation	T	Sig.(2 tailed)
Experimental	21	11.1429	4.95236	1.020	0.314
Control	22	9.5	5.56990		

#### Post-test

The mean scores of the experimental group and control group on post-WDT have been compared using an independent samples *t*-test. The findings, shown in Table 4, showed a statistically significant difference in the scores for the experimental group (Mean = 17.76, SD = 6.04) and the control group (Mean = 10.68, SD = 6.21) in favor of the experimental group. The *p*-value detected (*p* = 0.000) is smaller than the pre-determined significance level (*p* < 0.05). Hence, the values obtained for both groups revealed that there was a statistically significant difference. By implementing explicit conjunction instruction, the mean score of the experimental group increased significantly, indicating the potential for accelerated improvement in writing skills.

**TABLE 4:** Independent samples *t*-test for the difference in the mean scores of (WDT) post-test for the experimental group and the control group.

Group	Number	Mean	Std. deviation	T	Sig.(2 tailed)
Experimental	21	17.7619	6.04901	3.783	0.000
Control	22	10.6818	6.21355		

To determine whether the mean scores of each group had changed significantly after the intervention, paired sample *t*-tests were utilized, that is, measuring the differences between pre and post tests for each group. In Table 5, a comparison of the experimental group’s pre- and post-test results shows that students’ mean score increased significantly. In the pre-test, the mean score was (Mean = 11.14), while in the post-test, the mean score was (Mean = 17.76). The obtained *p*-value (*p* = 0.000) is smaller than the pre-determined significance level (*p* < 0.05), indicating a significant difference in the mean scores. These results support the previous findings and suggest the potential effectiveness of teaching conjunctions explicitly as cohesive devices (Abdelreheim, 2014; Adiantika, 2015; Boukra et al., 2019; Othman, 2019; Alqasham et al., 2021).

**TABLE 5:** Paired Samples *t*-test for the difference in the mean scores of (WDT) pre and post-test for the experimental group.

Group	Number	Mean	Std. deviation	T	Sig.(2 tailed)
Experimental pre-test	21	11.1429	4.95236	-8.462	0.000
Experimental post-test	21	17.7619	6.04901		

Despite a slight improvement, the control group’s post-test results indicate no statistical significance, as shown in Table 6. In the pre-test, the mean score was (Mean = 9.5), while in the post-test, the mean score was (Mean = 10.65). The obtained *p*-value (*p* = 0.069) is greater than the pre-determined significance level (*p* < 0.05).

**TABLE 6:** Paired Samples *t*-test for the difference in the mean scores of (WDT) pre and post-test for the control group.

Group	Number	Mean	Std. deviation	T	Sig.(2 tailed)
Control pre-test	22	9.5	5.56990	-1.919	0.069
Control post-test	22	10.6818	6.21355		

### Results of paragraph writing test

#### Pre-test

Table 7 presents the conjunctive ties used by participants in their pre-PWT, with the frequencies in each group as well as percentages of the four conjunction subcategories. Causative devices (34.72%) were the most used subclassification, followed by additive ties (31.94%), temporal ties (30.56%), and adversative ties (2.78%).

**TABLE 7:** Conjunctive ties used by students in their pre (PWT).

Type of conjunction	The conjunctive tie	Frequency in experimental group	Frequency in control group	Percentage
Additive	In addition	1	1	31.94%
	For example, For instance	3	0	
	And	2	0	
	Also	5	11	
Adversative	But	1	0	2.78%
	However	1	0	
Causal	Because	11	9	34.72%
	So	3	1	
	For these reasons	0	1	
Temporal	First, second, third.	3	0	30.56%
	In the beginning, to begin with	0	3	
	Then	2	0	
	Finally	3	5	
	In the end	2	1	
	Lastly	0	1	
	In short	2	0	
Total	72	39	33	100%

According to the pre- PWT results, participants had a limited understanding of conjunctive devices and could use some of them in their writing. Indeed, it is not surprising that students tended to find it easier to connect pieces of their writing with simple words rather than longer phrases since the majority of them (71%) were only basic users as mentioned earlier. Some students may have been unfamiliar with more complex conjunction devices or found them difficult to use. When it comes to additive devices, the most common cohesive tie was “also”, whereas items like “further” and “moreover” were never used. Among all forty-three students, the only adversative ties used were “but” and “however”, with each used only once. “Because” was the most frequently used causal tie, followed by “so”. Regarding temporal ties, students used the words “first”, “second”, “finally”, and “in the end” to indicate the order of their thinking more often than others.

Further, the results of the pre-PWT showed that Saudi EFL learners overused causative devices (34.72%) in contrast with other studies where causal devices ranked third (Abdelreheim, 2014; Aziz and Nuri, 2021) or even the fourth (Alshalan, 2019). The following script from a student’s paragraph shows that the causal item “because” was found to be repeated multiple times in a short script.

Example:

Paragraph 19: “I will choose pink color because it is associated with girly things. Also, I will choose blue because it expresses joy. The last color is violet because it is comfortable for eyes.”

Likewise, some students used the additive tie “also” inappropriately. Here is an example:

Paragraph 14: “If I start my own business, I prefer to open a beauty and care store. I will add a lot of lavender flowers all over the place. Also, I will choose the white color for the walls because I feel peace and calm. Also, I will add paintings.”

Another example of inappropriate use of the temporal tie “then” is shown here:

” …they can read digital books and search for information easily. Also, then classroom is available in Blackboard of Education.”

Although students’ showed use of the conductions (Table 7), analysis of their writing as just discussed above revealed the students’ poor knowledge and improper use of conjunctive devices. The students’ writing lacked cohesion and clear connection of ideas in a paragraph.

#### Post test

Table 8 shows that participants in both groups used more conjunctive ties in the post- PWT. However, the experimental group performed significantly better than the control group. The experimental group applied 124 ties, while the control group applied only 53.

**TABLE 8:** Conjunctive ties used by students in their post (PWT).

Type of conjunction	The conjunctive tie	Frequency in experimental group	Frequency in control group	Percentage
Additive	In addition	8	1	36.16%
	For example, For instance	14	6	
	And	7	7	
	Besides	3	0	
	In other words	2	0	
	Moreover	2	0	
	Or, or even	2	0	
	Furthermore	1	0	
	Plus	1	0	
	Also	5	2	
	I mean	0	1	
	In the same way	1	0	
	Similarly	1	0	
Adversative	But	4	3	9.60%
	However	4	0	
	Not only but also	1	0	
	Instead	0	1	
	On the other hand	3	0	
	In fact	1	0	
Causal	Because	11	9	22.60%
	So	6	6	
	As a result	3	0	
	Therefore	2	0	
	Due to	1	0	
	For these reasons	2	0	
Temporal	First, second, third.	21	9	31.64%
	Then	1	1	
	Finally	6	3	
	In the end	2	1	
	Today, nowadays	2	1	
	In this time	0	1	
	Briefly	1	0	
	In the beginning	0	1	
	In short	2	0	
	In conclusion	3	0	
	To sum up	1	0	
Total	177	124	53	100%

Despite the distinctions between the four types, learners adopted 177 different devices in total. They depended most heavily on additives, as they represent (36%) of the total cohesive relations generated. Temporal is ranked second (31%), followed by causal (22%), while adversatives are ranked fourth (9.6%). Regarding the ranking of the categories, these findings concur with Abdelreheim’s (2014) study, but contradict with Aziz and Nuri’s (2021) findings, which indicated that temporal was the most frequently used category by Kurd Iraqi learners. However, the findings of these studies and the present study show that adversative ties were scarcely employed. One possible interpretation is that a text’s type could affect the percentages attributed to conjunction subclassifications, as suggested by Abdelreheim (2014) and Afzaal and Xiangyi (2020).

Table 8 indicates that *for example* and *for instance* were the most frequently used additive devices followed by *in addition*. This might be attributed to the text nature as the students were required to respond to the following question “*How do companies become popular*” and they provided several examples to support their ideas. The items *and* and *also* came next. It is thought that the students were familiar with employing these items more than others. Various additive items such as: *besides, in other words, moreover, plus, furthermore*, and comparative devices such as: *in the same way, similarly* were also employed in the writings produced by the experimental group students. Regarding the adversative devices, the learners depended mostly on the item *but* to establish an adversative relation between their sentences. This could be because the item *but* is more common than other items. The item, *however*, was also employed four times by the experimental group students. They were able to employ other items including*: on the other hand* and *in fact.*

In order to establish a causal relationship between their sentences, students tended to use the words *because* followed by *so*. Obviously, these items were more prevalent than others. In the experimental group, students were able to employ other items such as: therefore, for these reasons, as a result *and* due to appropriately after the treatment. Furthermore, Table 8 demonstrates that students used temporal items *first, second, third* and *finally* the most. This indicates students’ comprehension of how to begin and end their paragraphs. By establishing a cohesive relation between the sentences in the texts, readers can anticipate the inclusion of other points or ideas. Several temporal items that indicate summarizing such as: briefly, to sum up, and in short were employed by students in experimental group.

As can be seen from the results discussed above, results from the post- PWT supported the post- WDT results, as students in the experimental group were able to successfully and more frequently identify the relationship between sentences in their paragraphs and determine the appropriate item to link the sentences. This benefits the reader in connecting parts of the paragraph. Analysis of students’ compositions in the post-WDT showed that they seemed to be more cohesive, unified, and easier to read, encouraging the reader to explore the writer’s ideas as the message swiftly move from one thought to another. This finding concurs with Adiantika (2015) and Alqasham et al. (2021).

Furthermore, the students were able to display a variety of conjunctive items instead of relying solely on simple ones. This makes their paragraphs more interesting for the reader. Prior to the experiment, items such as “I mean”, “in other words”, “on the other hand”, and “as a result” were difficult to find. Here are some samples of the students’ composition after being taught conjunctions explicitly:

Example 1:

Paragraph (13) “… we always hear about the competition between Pepsi and Coca-Cola. In KSA, we prefer Pepsi more than Coca-Cola. On the other hand, Brazilian people prefer Coca-Cola over Pepsi, and this is because of the marketing.”

Example 2:

Paragraph (12) “…. One example of a popular company is Uber, the main reason of its popularity is the good service they offer, and where they started. Besides offering a special service, going viral helps a lot. For instance, flix’s jump made a soft drink company popular.”

Example 3:

Paragraph (17) “… We may expect that the great succession in companies is due to the products. In fact, it is a relatively correct idea, however, there are basics and strategies.”

Example 4:

Paragraph (15) “… To sum up, doing something surprising is a good way to make a product popular.”

The control group, in contrast, continued to use simple conjunctive items. With a slight increase in the frequency.

As can be seen above, the results of both post- WDT and post- PWT showed a remarkable improvement in favor of the experimental group. The students’ mean scores changed significantly following explicit instruction. Not only did students’ scores increase, but also the quality of their writing. The students were able to compose more cohesive, unified, and interesting paragraphs despite the fact that the majority of the learners in the present study were beginners. It seemed that students’ competence to employ cohesive devices effectively increased by conscious understanding of their forms and implications. The findings of the present study confirmed Alshalan (2019) who maintained that using cohesive devices in writing can be one indicator of high-quality writing. This use, however, must be appropriate in order to increase the quality of the paragraph.

Conversely, the results of the post-test for the control group were almost the same as the pre-test regarding WDT. Moreover, even while the post-test findings for PWT showed a slight increase in the quantity of conjunctive items utilized, they remained much lower than that of the experimental group. Besides, students in the control group continued to employ simple items regularly.

Based on the discussion above, the experimental group participants made significant progress in paragraph writing, and this is believed to be due to the explicit instruction of conjunctions, especially in terms of cohesion.

## Conclusion

The primary goal of the present study was to investigate the effectiveness of explicitly teaching conjunctions as cohesive devices on enhancing students’ paragraph writing. In order to achieve this objective, an experiment design with pre- and post-tests was used, and data was analyzed both quantitatively and qualitatively. The present study concluded that the intervention that was employed had a positive effect on students’ performance. Cohesion, as one indicator of effective writing, was achieved to through the use of conjunctions taken into consideration the low level of proficiency of the population. The post-test results of WDT revealed that the mean scores of the experimental and control groups were statistically significantly different, suggesting that the students of the former have remarkably improved in terms of comprehension and application of conjunctions as part of the cohesiveness concept. Moreover, the results of post- PWT revealed that the experimental group successfully identified the relationship between sentences in their paragraphs and, for the most part, chose the appropriate item to connect the sentences.

The current study has provided an important insight by exploring the role of explicit instruction of conjunctions on developing students’ writing. Such instruction might contribute to a development in the context of teaching as well as learning writing, especially that previous studies have demonstrated that EFL learners struggle with the use of cohesive devices, particularly conjunctions (Arabi and Amin Ali, 2014; Othman, 2019; Alqasham et al., 2021).

It is recommended that instructors be encouraged to explicitly teach EFL students how to use conjunctions appropriately to develop their paragraph writing. Designers of textbooks should also be aware of the advantages of integrating such devices into writing classes and assign tasks that call for their use. Needless to say, although the present study has shed some light on an underrepresented population, it is suggested that research on larger samples and at different levels of study can be conducted to generalize the results. Also, the current study was confined to conjunctions as a predictor of cohesion in students’ writing. More studies might be done to look at additional types of cohesiveness and uses such as complementary use of conjunctions.

## Data availability statement

The raw data supporting the conclusions of this article will be made available by the authors, without undue reservation.

## Ethics statement

Ethical review and approval was not required for the study on human participants in accordance with the local legislation and institutional requirements. Written informed consent for participation was not required for this study in accordance with the national legislation and the institutional requirements.

## Author contributions

Both authors listed have made a substantial, direct, and intellectual contribution to the work, and approved it for publication.
